# Primary Hepatic Neuroendocrine Tumor: A Case Report and Literature Review

**DOI:** 10.1155/2024/9181560

**Published:** 2024-02-26

**Authors:** Souad Ghattas, Jad Al Bitar, Georges Chahine, Francois Kamar, Marwan Haddad, Raja Wakim

**Affiliations:** ^1^Department of General Surgery, Mount Lebanon Hospital University Medical Center, University of Balamand, Beirut, Lebanon; ^2^Department of Oncology, Mount Lebanon Hospital University Medical Center, University of Balamand, Beirut, Lebanon; ^3^Departement of Radiology, Mount Lebanon Hospital University Medical Center, University of Balamand, Beirut, Lebanon

## Abstract

Primary hepatic neuroendocrine tumors (PHNETs) are extremely rare and account for about 0.3% of all neuroendocrine tumor cases. Resection is usually difficult because they are usually diagnosed in the late stages. We report the case of a patient diagnosed with PHNETs, initially classified as unresectable but then underwent a successful left hepatectomy. PHNETs are rare malignant tumors, and a high index of suspicion is warranted for the diagnosis after excluding the presence of a primary extrahepatic lesion. Radical hepatectomy can be curative when feasible along with a combination of multiple treatments that improve the prognosis.

## 1. Introduction

NETs are rare type of tumor, deriving from the cells of the neuroendocrine system [1]. They are usually found in the gastrointestinal tract (50%) or the bronchopulmonary tree (30%) [2]. For the liver involvement in NETs, it is usually metastasis from other sites. PHNETs are extremely rare, leading to a difficult diagnosis preoperatively [3]. The diagnosis of PHNETs is made based on an immunohistochemistry compatibility with NET and exclusion of metastases from other sites [4]. Resection of PHNETs is usually difficult because they are diagnosed in late stages when the tumor has already grown larger and invaded vital structures [5]. Herein, we report the case of a patient diagnosed with PHNETs, initially classified as unresectable but then underwent a successful left hepatectomy.

## 2. Case

This is the case of a 55-year-old male patient with negative past medical and past surgical history. History of this patient goes back to 2019 when he first sought medical care for chronic diarrhea of more than 8 episodes per day and decrease PO intake. The patient denied a history of jaundice, vomiting, or flushing. Physical examination showed a soft abdomen with no palpable masses. Laboratory tests were within the normal range. Computed tomography (CT) scan of the abdomen showed a 4 cm left liver mass. Further assessment of this mass with magnetic resonance imaging (MRI) revealed a 4.5 cm lesion at the central aspect of the left lobe of the liver arising or pointing towards the liver hilum, extending among segments II, III, and IV. Lesion was encasing the left main portal vein with even focal suspicion of a very minimal intravenous extension noted on one cut at approximately the origin of the left portal vein. There was a high degree of suspicion of occlusion of the left hepatic vein reaching but not extending into the inferior vena cava. Tumors markers CA 19-9 and alpha fetoprotein (AFP) returned negative. Ultrasound liver biopsy was performed for a conclusive diagnosis. Histopathological examination showed the presence of metastatic tumor consistent with well-differentiated neuroendocrine tumor grade 2. Tumor cells were positive for cytokeratin, CK 20, synaptophysin, and CDX2. Index of proliferation as assessed by Ki 67 was approximately 5%. To rule out the presence of metastasis from a primary lesion outside the liver, further gastroscopy and colonoscopy were performed and no extrahepatic lesions were found. Fluorodeoxyglucose positron emission tomography-CT (PET-CT) showed an ill-defined subtle hypodense area with adjacent ductal prominence seen in segment III of the liver showing minimal enhancement and negligible uptake measuring 3.9 × 3.4 cm.

Our patient was diagnosed with primary hepatic neuroendocrine tumor and sought medical care in India in 2019. He was not considered to be a surgical candidate and instead, he was started on chemotherapy. He received 4 sessions of chemotherapy with etoposide and cisplatin. A follow-up PET-CT showed partial response, decrease of the size of the lesion to 3.2 × 3.5 cm. He then received 4 additional sessions of chemotherapy and repeat PET-CT showed further decrease in the size of the lesion, reaching 2.2 × 1.9 cm.

Patient stopped treatment until 2021 when a repeat PET-CT showed the previously described ill-defined hypodense lesion in the left liver lobe, mainly seen in segment III with extension to segment IV and II, measuring 2.2 × 1.9 cm. But a new 4.1 × 3.1 cm low grade FDG avid lobulated soft tissue lesion in the porta hepatis which seems encasing the portal vein was also seen. Gallium-DOTATATE whole body scan showed a 5.0 × 6.3 cm abnormal soft tissue mass lesion at the porta hepatis; this mass infiltrates the caudate lobe and hepatic segment IV b and a lesion infiltrating the hepatic segments II, III, and IV, measuring approximately 5.8 × 6.7 cm.

**Figure 1:**
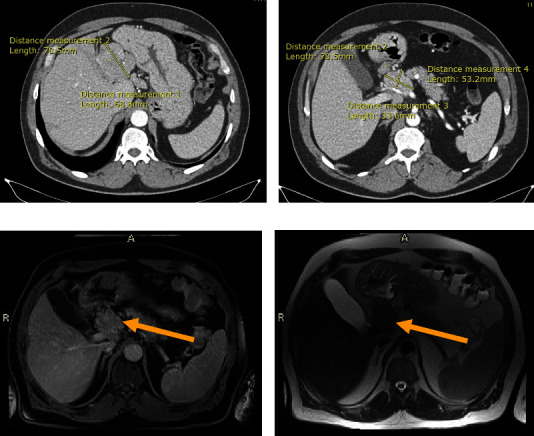
(a) Enhanced CT scan transversal view showing 6.8 × 5.1 left liver lobe mass. (b) Enhanced CT scan transversal view showing 5.3 × 3.3 lesion encasing the hepatic artery without obstructing it. (c) MRI showing 45 × 47 mm multinodular lesion hypo T1 diffusion restricting the lesion at the porta hepatis (yellow Arrow). (d) MRI showing 45 × 47 mm multinodular lesion hyper T2 diffusion restricting the lesion at the porta hepatis (yellow Arrow).

The patient presented to our hospital in July 2022, and repeat CT scan showed 5.3 × 3.3 lesion at the porta hepatis encasing the hepatic artery without occluding it, along with another 6.8 × 5.1 left liver lobe mass. The masses were better assessed using magnetic resonance imaging (MRI), showing the atrophic left lobe of the liver being occupied by a hypo T1 and hyper T2 diffusion restricting lesion measuring 53 × 51 × 71 mm, resulting in focal dilatation of the intrahepatic biliary ducts. There is a hypo enhancement after the administration of gadolinium, raising the possibility of poorly differentiated tumor. At the porta hepatis, there is a 45 × 47 mm multinodular lesion distinct from the pancreas and adjacent structures. It engulfs but does not invade the hepatic artery. Findings likely represent a tumor extension versus matted lymph nodes. The patient received 4 new cycles of chemotherapy with dacarbazine and 5FU. Then, repeat CT scan showed unchanged liver and porta hepatis masses (Figure 1).

**Figure 2:**
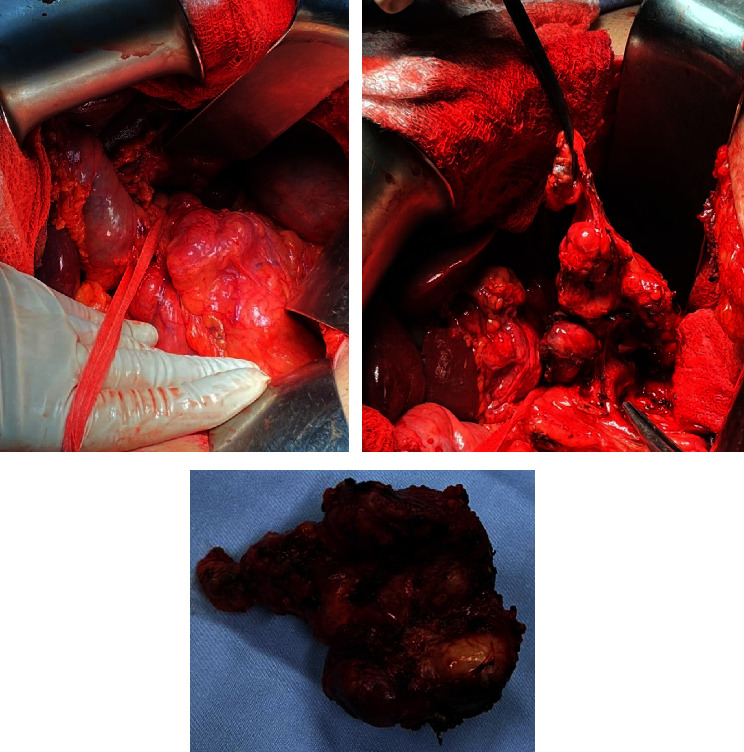
Large lymph node at the level of the porta hepatis and common hepatic artery removed en bloc.

**Figure 3:**
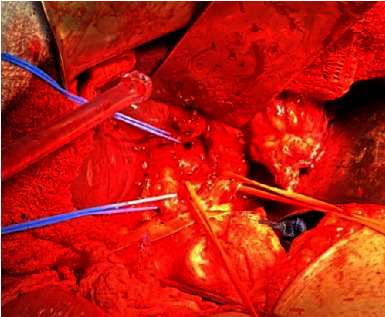
Isolation using vessel loops of the common bile duct, right and left ducts, common hepatic artery, right and left, and the portal vein, right and left.

**Figure 4:**
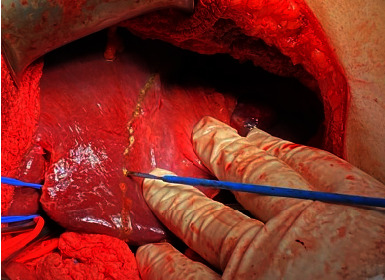
Demarcation line between the right and left lobes.

**Figure 5:**
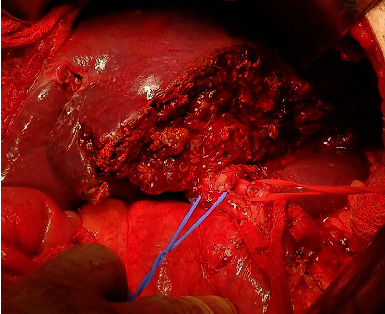
Under vision ligation and divisions of the left hepatic artery, left portal vein, and left bile duct.

**Figure 6:**
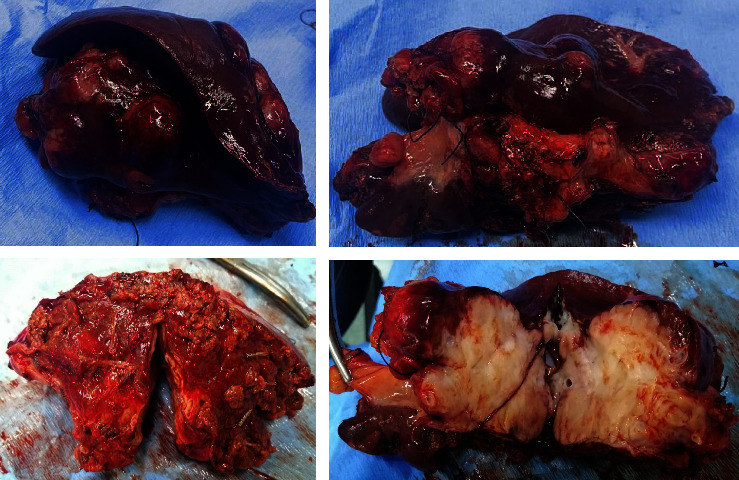
Specimen removed and opened.

Decision was made to proceed with surgical management. Access to the abdomen was done through a right subcostal incision, left liver mass was identified very adherent to the lesser omentum, and delicate adhesiolysis was done till the identification of a large lymph node at the level of the porta hepatis and common hepatic artery (Figure 2). Delicate and complete dissection of the lymph nodes en bloc that was abutting the common hepatic artery was done. Then, a cholecystectomy and mobilization of the left liver was done by dissecting all its attachments. Common bile duct, right and left ducts, common hepatic artery, right and left, and the portal vein, right and left, were all identified and isolated using vessel loops (Figure 3). Selective clamping of the left lobe was done by controlling the left hepatic artery and left portal vein using bulldogs. The demarcation line between the right and left lobes was seen (Figure 4). The line was marked on the liver using electrocautery. Dissection started caudally to cranially using Kelly and bipolar. All further ligation and divisions were done under vision (site of entrance to the left lobe) (Figure 5). The left hepatic artery, left portal vein, and left bile duct were all ligated and divided. The middle hepatic vein was identified and preserved. After full dissection of the left liver lobe, control of the left hepatic vein was done using GIA purple, and the resected specime was removed (Figure 6). Additional hemostasis was done by placement of surgical. A lamellated drain was left at the surgical bed. Surgery time was 5 hours, blood loss 300 cc, and no pringle maneuver was used. Final histopathology result and immunohistochemical study confirmed the diagnosis of a well-differentiated neuroendocrine tumor of the liver, classified as grade 2. The tumoral cells expressed CD 56, chromogranin, and synaptophysin in an intense and diffuse manner. Proliferation index ki67% is estimated around 8%. The pathology result confirmed the presence of an intact capsule, negative resections margins, and presence of vascular and perineural invasion with 4 positive lymph nodes metastasis over 4 resected.

On postoperative day 1, the patient was dyspneic and tachypneic. A CT chest pulmonary embolism (PE) protocol was done and the result showed the presence of bilateral PE. The patient was started on therapeutic anticoagulation. He received two units of blood on postoperative. Diet started on day 2 postoperative well tolerated and progressed. Laboratory studies were stable and showed no liver failure. The drain output was serosanguinous and minimal throughout the stay, removed on day 5 postoperative, and patient was discharged home.

**Figure 7:**
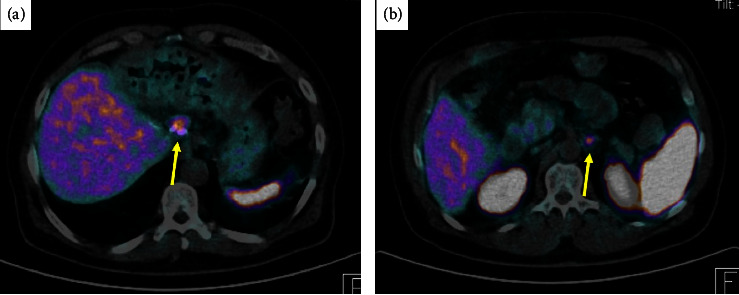
Gallium-DOTATATE whole body transversal view showing (a) right para gastric lymph node 2.6 × 1.4 cm SUV max 8.9 (yellow arrow) and (b) left para-aortic lymph node 1.2 × 0.9 cm SUV max 7.2 (yellow arrow).

On the follow-up after one month, the patient was clinically doing well, denies any diarrhea, and imaging showed no new appearing lesions. He received 3 cycles of adjuvant chemotherapy, and a follow-up Gallium- DOTATATE whole body scan 3 months after the surgery showed newly appearing lymph nodes metastasis, a right para gastric lymph node 2.6 × 1.4 cm SUV max 8.9, and another left para-aortic lymph node 1.2 × 0.9 cm SUV max 7.2 (Figure 7). Then, the patient received 5 cycles of chemotherapy. Follow-up imaging showed stable para gastric and left para-aortic lymph nodes.

**Figure 8:**
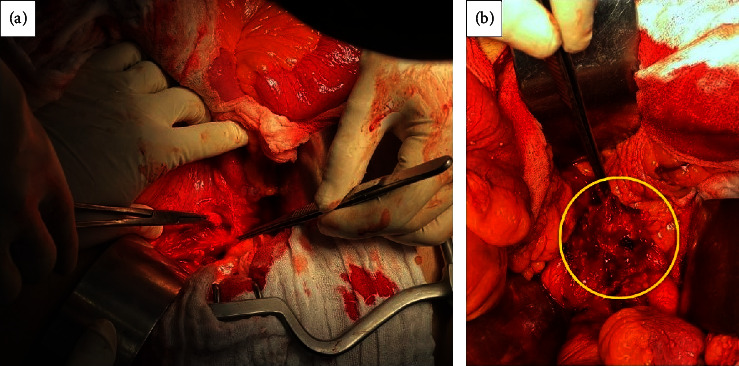
Intraoperative pictures showing (a) left para-aortic lymph nodes and (b) gastrohepatic lymph nodes (yellow circle).

At this time, one year after the hepatectomy, a multidisciplinary meeting was held and decision was taken to proceed with debulking surgery. Excision of the left para-aortic, celiac, and gastrohepatic lymph nodes was done (Figure 8). The final pathology result was consistent with multiple metastasis of the known neuroendocrine tumor; gastrohepatic lymph node 1+/2, left para-aortic 4+/6, hepatic hilium 3+/5, and celiac lymph nodes 2+/2.

The postoperative course was uneventful and the patient was discharged home on day 3 postoperatively. In the follow-up 3 months after the surgery, patient denies any symptoms and follow-up imaging negative for any recurrent disease.

## 3. Discussion

NETs are very rare malignant tumors accounting for about 1%-2% of all gastrointestinal tumor cases. Liver metastases are common in NETs but PHNETs are extremely rare, accounting for about 0.3% of all neuroendocrine tumor cases [6].

PHNETs are very slow growing tumor [6]. Also, due to the rarity, little is known about it. The liver contains only a small number of neuroendocrine cells, so the pathogenesis of PHNET is still unclear but multiple hypotheses about its origin have been proposed due to the multifunctional stem cells of the liver or other ectopic tissues with endocrine functions [7].

PHNETs occur more in the middle aged and elderly patients with a median age of 52–63 years [8]. It is not sex specific and most patients present with symptoms but the presentations tend to be nonspecific with abdominal pain being the most common complaint [8]. PHNETs differ from other NETs as they are mostly nonfunctional, there is no carcinoid syndrome manifested. Only 10% of the cases of PHNET present with the triad of abdominal pain, skin flushing, or diarrhea [8]. This helps us differentiate NET metastasis to the liver from other origin because in these cases, greater than 50% of the patients have carcinoid syndrome [8]. When the tumors are discovered, it had reached an advanced stage with a large tumor size, consequently leading to delay treatment [7]. Our case had a functional PHNET and presented with multiple episodes of diarrhea. PHNETs have not been found to be related to liver cirrhosis or hepatitis [2].

Clinical tumor markers AFP-CEA and CA 19-9 have no diagnostic value in PHNET. But neuron specific enolase (NSE), CgA, 5-HIAA, and Syn were more effective in the pathological diagnosis of PHNETs [9].

The WHO classification of neuroendocrine tumors of the digestive system (2019) divides NETs into three grades based on the number of mitoses per 10 high power microscopic fields or the percentage of neoplastic cells immunolabeled for the proliferation marker Ki67 [10]. Our patient was classified as well-differentiated neuroendocrine tumor grade 2, with a proliferation index ki67 estimated around 8%.

The diagnosis of PHNET is based on two prerequisites. The liver mass must be immunohistochemically compatible with NET, along with the absence of clinical, endoscopic, or imaging findings of another site of origin for the NET [9].

Medical imaging examinations such as ultrasound, CT scan, and MRI have a low sensitivity and specificity for diagnosis of PHNET [11]. The role of imaging is primarily in the identification of hepatic lesions and aiding in the search for a possible primary in the intestinal tract, pancreas, or other organs [11]. Gallium-68 DOTA PET-CT has a sensitivity of 93% and specificity of 91% in identifying the tumor in gastroenteropancreatic NETs [11]. The absence of radiotracer avid disease elsewhere or the presence of low-volume disease in specific locations such as upper abdominal lymph nodes or osseous structures would, therefore, be highly suggestive of PHNET [11].

Pathological diagnosis based on histological and immunohistochemical evaluation is regarded as the final diagnosis standard for PHNETs [12]. In our case, histopathology did not directly diagnose PHNETs but PET/CT showed only the presence of a hepatic mass, which was valuable in diagnosing PHNETs. Long-term follow-up is essential to search for extrahepatic primary.

Extrahepatic metastatic disease is reported in about 20% of the patients at the time of presentation, with the bone, lymph nodes, and lungs being the most common sites of spread [13]. In our case, the patient had progression of his disease with new appearing porta hepatis metastatic lymph nodes when he stopped treatment.

Surgical resection is generally the preferred treatment for PHNETs and R0 excision can be curative [3]. The success rate of PHNET liver resection has been reported to be approximately 70% and the 5-year survival rate after surgery is as high as 78% [1]. For patients with unresectable disease, various palliative options exist, but the role and effectiveness of these treatment modalities remain unclear and need further study [9]. They can improve survival and optimize for resection, such as systemic chemotherapy, and the cytotoxic drugs are a good choice for tumors with a high proliferation index, but the most highly recommended protocol involves a combination of 5-fluorouracil (5-FU) and etoposide [9]. Hepatic transarterial chemo-embolization (TACE), somatostatin analogues, radiofrequency ablation, and percutaneous ethanol injection treatment are other treatment modalities for unresectable disease [5]. Liver transplantation has been suggested to be a treatment option in selected patients with multiples lesions or impaired liver function [5]. Our patient first had downstaging of the tumor with systemic chemotherapy, but the tumor progressed after the treatment was stopped with a new appearing mass at the porta hepatis; therefore, surgery was the optimal treatment in our case.

The recurrence rates of PHNETs after surgery were as high as 20–40% [9]. Our patient was diagnosed with lymph nodes metastasis only 3 months after the initial surgery and while he was on adjuvant chemotherapy. Therefore, a close follow-up is recommended in the postoperative period [9].

## 4. Conclusion

PHNETs are extremely rare malignant tumor and often misdiagnosed. A high index of suspicion is warranted and the diagnosis should be considered after excluding the presence of a primary extrahepatic lesion. There are no treatment guidelines for PHNETs but radical hepatectomy can be curative when feasible and combination of multiples treatments can be used to improve the prognosis. A close follow-up is recommended in the postoperative period.
